# Cardiopulmonary Failure in Hantavirus Disease: Mechanisms, Recognition, and ECMO-Based Management

**DOI:** 10.3390/v18080915

**Published:** 2026-08-20

**Authors:** Deng Siang Lee, Aboubakr Hasan

**Affiliations:** 1 Faculty of Medicine and Health Sciences, University of Barcelona, 08007 Barcelona, Spain; 2 Department of Cardiothoracic Surgery, Stanford University, Stanford, CA 94305, USA; aboubakr@stanford.edu

**Keywords:** hantavirus, hantavirus pulmonary syndrome, hantavirus cardiopulmonary syndrome, Sin Nombre virus, Andes virus, extracorporeal membrane oxygenation

## Abstract

**Background:** Hantavirus pulmonary syndrome (HPS), also designated hantavirus cardiopulmonary syndrome, is caused by New World hantaviruses, principally Sin Nombre virus in North America and Andes virus in South America. The syndrome is characterized by rapidly progressive noncardiogenic pulmonary edema and myocardial depression, with case fatality rates of 25% to 40%. A 2026 outbreak aboard an expedition cruise ship in the South Atlantic, comprising 13 cases and three deaths, confirmed that Andes virus can be transmitted between humans in a confined setting remote from the rodent reservoir. **Methods:** Virological, pathophysiological, clinical, and therapeutic aspects of HPS were reviewed, with particular emphasis on cardiopulmonary mechanisms. Sources were identified through PubMed, Scopus, and Google Scholar, with priority given to original research articles, clinical series, and controlled trials published through 2025. Literature published in English and Spanish was included. **Results:** Pathogenic hantaviruses enter endothelial cells and platelets via αvβ3 integrins, disrupting the VEGF-VEGFR2 signaling axis and rendering endothelial cells hypersensitive to physiological VEGF concentrations. Expansion of CD8+ T cells and activated macrophages releases TNF-alpha, IFN-gamma, and nitric oxide, amplifying microvascular permeability and contributing to myocardial depression. Autopsy studies demonstrate direct hantaviral myocarditis with viral antigen in cardiac endothelium and interstitial macrophages. Transpulmonary thermodilution confirms simultaneous hypovolemia, reduced global ejection fraction, and elevated extravascular lung water. Because the incubation period is long and the cardiopulmonary phase is substantially immune-mediated, seroconversion precedes rather than follows clinical deterioration, which preserves the diagnostic utility of IgM serology in a disease that can kill within 48 h. VA-ECMO initiated at the first signs of cardiopulmonary decompensation has reported survival rates approaching 80% in selected experienced centers. No antiviral has demonstrated efficacy in controlled trials during the cardiopulmonary phase, and no licensed vaccine exists. **Conclusions:** HPS produces a mixed shock state through increased microvascular permeability, T cell-mediated immunopathology, and direct myocarditis. Management follows a stepwise algorithm: suspected HPS triggers immediate complete blood count with peripheral blood smear and concurrent hantavirus IgM serology and RT-PCR, followed by ICU admission, conservative fluid resuscitation guided by transpulmonary thermodilution, and early contact with an ECMO-capable center at the first sign of rising lactate, falling cardiac index, refractory shock, arrhythmia, or rapid oxygenation failure.

## 1. Introduction

In May 1993, a cluster of previously healthy young adults in the Four Corners region of the United States presented with acute respiratory failure from an unrecognized cause. Retrospective surveillance identified 24 cases since December 1992, with a case fatality rate of 50% [1]. The clinical series of the first 17 confirmed patients, published in the *New England Journal of Medicine* in 1994, reported a 76% case fatality rate within that cohort [2]. The causative agent was identified as Sin Nombre virus (SNV), an enveloped, negative-sense, tripartite RNA virus of the genus *Orthohantavirus*, family Hantaviridae [3]. In South America, Andes virus (ANDV) produces an identical syndrome and is the only hantavirus with documented person-to-person transmission [4]. The United States has reported 890 confirmed cases from 1993 through 2023, averaging fewer than 30 per year, with the highest burden in New Mexico, Colorado, and Arizona [5]. In South America, more than 100 cases occur annually; Argentina consistently reports the highest regional case counts [6]. Across eight countries in the Americas in 2025, 229 cases and 59 deaths were recorded (case fatality rate 25.7%) [6]. Transmission is via inhalation of aerosolized viral particles from the excreta, urine, or saliva of infected rodents. *Peromyscus sonoriensis* (western deer mouse) is the primary reservoir for SNV in North America; *Oligoryzomys longicaudatus* (long-tailed pygmy rice rat) is the primary reservoir for ANDV in southern South America [7,8].

Hantaviruses have a trisegmented, single-stranded, negative-sense RNA genome of approximately 11.5 to 12 kb in total. The small (S; approximately 1.6–2.1 kb), medium (M; approximately 3.6–3.7 kb), and large (L; approximately 6.5 kb) segments encode the nucleocapsid (N) protein of approximately 428 amino acids, the glycoprotein precursor (co-translationally cleaved at a conserved WAASA motif into the surface glycoproteins Gn and Gc), and the RNA-dependent RNA polymerase of approximately 2150 amino acids, respectively [3]. The genome therefore comprises four canonical open reading frames encoding four structural proteins. Several species, including Puumala, Tula, and Andes viruses, additionally encode a non-structural NSs protein from an overlapping +1 reading frame within the S segment, which antagonizes type I interferon induction [9]. Each segment carries complementary 5′ and 3′ terminal sequences that base-pair to form a panhandle structure, a feature directly relevant to innate immune recognition (Section 4.2). Hantaviruses are not arthropod-borne; human infection requires direct exposure to aerosolized rodent excreta.

HPS has since been recorded far outside its endemic range, in circumstances that bear directly on the clinical themes of this review. On 27 April 2026 a passenger was medically evacuated from the Netherlands-flagged expedition cruise ship MV Hondius, which had sailed from Ushuaia, Argentina, on 1 April 2026 and called at Antarctica, South Georgia, Tristan da Cunha, Saint Helena and Ascension Island; he had severe acute respiratory infection progressing to shock and acute respiratory distress syndrome, and hantavirus infection was confirmed by pan-hantavirus RT-PCR at the National Institute for Communicable Diseases in South Africa on 2 May 2026, with sequencing of the L segment identifying ANDV on 5 May [10]. By the time the World Health Organization declared the outbreak over on 2 July 2026, 13 cases (12 laboratory-confirmed and one probable) and three deaths had been notified, a case fatality ratio of 23%; the median age of cases was 65 years (interquartile range 56 to 70 years) and the three fatal cases were aged 69, 70 and 79 years [11]. Epidemiological and genomic evidence indicates a point-source introduction, most probably acquired on land before embarkation, followed by limited chains of person-to-person transmission aboard the vessel without sustained onward spread; contact tracing was undertaken in 33 countries and territories, and 317 high-risk contacts completed the 42-day monitoring period without further cases being detected [11,12]. Three features of this episode are pertinent to what follows. First, it demonstrates that ANDV can propagate in a confined setting thousands of kilometres from the rodent reservoir, so that geographical distance from an endemic region does not exclude the diagnosis. Second, the syndrome presented to clinicians without previous experience of it and without local confirmatory testing, and the initial differential diagnosis was directed towards atypical pneumonia, sepsis, malaria and dengue [10]; the peripheral blood smear criteria and the diagnostic sequence set out in Section 6 are intended for exactly this situation. Third, management rested entirely on supportive critical care, since no licensed antiviral or vaccine was available to the response, which is the position described in Section 7.4 and Section 7.6.

Hantavirus pulmonary syndrome (HPS), also known as hantavirus cardiopulmonary syndrome (HCPS), is distinguished from other viral pneumonias by the combination of increased microvascular permeability without direct endothelial cytopathic injury, superimposed immunopathology, hemostatic dysregulation, and myocardial depression. No antiviral has proven effective, and management remains supportive. No licensed vaccine is available, for reasons discussed in Section 7.6. This review synthesizes the mechanisms of cardiopulmonary failure in HPS and translates these into clinical practice.

## 2. Methods

Sources of the current narrative review were identified through a targeted search of PubMed, Scopus, and Google Scholar using the terms hantavirus, hantavirus pulmonary syndrome, hantavirus cardiopulmonary syndrome, Sin Nombre virus, Andes virus, vascular permeability, myocarditis, and extracorporeal membrane oxygenation. Original research articles, clinical series, and controlled trials were prioritized. Surveillance data were obtained from the Centers for Disease Control and Prevention and the Pan American Health Organization. English- and Spanish-language literature was included.

A record was eligible if it met at least one of the following: (i) it reported original human data on the pathophysiology, clinical course, diagnosis or treatment of HPS or HCPS; (ii) it was a randomised or otherwise controlled trial of an intervention in hantavirus disease; (iii) it reported mechanistic experimental work bearing directly on microvascular permeability, innate or adaptive immune responses, or myocardial injury in hantavirus infection; (iv) it was an autopsy or histopathological series; or (v) it was a surveillance report, outbreak investigation or clinical guidance document issued by a national or international public health authority. Records were excluded if they addressed hemorrhagic fever with renal syndrome without content transferable to HPS, if they were single case reports adding no mechanistic or management point not already represented.

Where several publications supported the same point, precedence was given, in descending order, to controlled trials, prospective human cohorts, autopsy and histopathological series, mechanistic laboratory studies, and official surveillance data; narrative reviews were cited only where they supply a synthesis not otherwise available, and secondary sources were replaced by the primary reports wherever those could be obtained. Reference lists of included articles were hand-searched for sources not retrieved electronically. Surveillance and outbreak data were taken from the Centers for Disease Control and Prevention, the Pan American Health Organization, the European Centre for Disease Prevention and Control and the World Health Organization. The specific basis on which each individual reference was included is detailed in Supplementary Table S1.

## 3. Viral Entry and Cellular Tropism

Pathogenic New World hantaviruses (SNV, ANDV, New York-1 virus) enter endothelial cells, platelets, and macrophages via αvβ3 integrins; the nonpathogenic Prospect Hill virus uses β1 integrins instead [13]. Pathogenic Old World hantaviruses causing hemorrhagic fever with renal syndrome (Hantaan, Seoul, Puumala viruses) also use β3 integrins [14]. This receptor specificity has direct pathogenic consequences: β3 integrins regulate vascular permeability and platelet activation, and their occupation by pathogenic hantaviruses disrupts both functions [13,14]. Protocadherin-1 has subsequently been identified as an essential entry factor for New World hantaviruses in pulmonary endothelial cells, providing a molecular basis for the pulmonary tropism that distinguishes HPS from hemorrhagic fever with renal syndrome [15]. Hantaviruses replicate extensively in pulmonary endothelial cells without inducing recognizable cytopathic changes; infected endothelial monolayers remain intact in vitro, and endothelial necrosis is absent at autopsy [16].

## 4. Pathophysiology of Cardiopulmonary Failure

### 4.1. Increased Microvascular Permeability and Pulmonary Edema

Following inhalation, SNV is taken up by alveolar macrophages, disseminates hematogenously, and replicates in pulmonary capillary endothelial cells without producing direct endothelial injury [16,17]. Increased permeability requires the combination of β3 integrin dysregulation and available VEGF. Pathogenic hantaviruses inhibit αvβ3 integrin function two to three days after infection, disrupting the αvβ3-VEGFR2 receptor complex that normally restrains VEGF-directed permeabilizing signaling [18]. In vitro studies with Hantaan, ANDV, and New York-1 viruses demonstrate markedly enhanced endothelial permeability in response to VEGF following infection, an effect absent with the nonpathogenic Prospect Hill and Tula viruses [18]. Angiopoietin-1 and sphingosine 1-phosphate suppress this hantavirus-directed permeability at physiological concentrations, as do antibodies to VEGFR2, identifying potential therapeutic targets [18].

Extravasation of protein-rich fluid into the pulmonary interstitium and alveoli produces the noncardiogenic pulmonary edema that defines HPS. Transpulmonary thermodilution in mechanically ventilated HCPS patients demonstrates markedly elevated extravascular lung water index (EVLWI) and pulmonary vascular permeability index (PVPI), confirming a permeability rather than hydrostatic mechanism [19]. EVLWI correlates inversely with global ejection fraction (r = −0.36) and mean arterial pressure (r = −0.27), indicating that pulmonary flooding directly tracks hemodynamic deterioration [19]. The resulting intravascular volume depletion independently contributes to the shock state.

The contribution of the pulmonary epithelium to increased alveolar permeability has been underexplored. A 2023 cross-sectional study measured plasma soluble receptor for advanced glycation end-products (sRAGE), a marker of type I alveolar epithelial cell injury, in critically ill HCPS patients and found substantially higher concentrations in severe compared with mild disease [20]. Whether this reflects a primary pathological event or a downstream consequence of severe hypoxia requires prospective study in larger cohorts.

### 4.2. Innate Recognition and Immunopathogenesis

Hantaviral RNA is sensed principally by the cytosolic RIG-I-like receptor RIG-I (DDX58), which recognizes the double-stranded panhandle formed by the complementary genomic termini [21]; TLR3 contributes to the response to Hantaan virus [22], and a role for TLR4 in sensing viral glycoproteins has been proposed. Recognition of hantaviruses has two features that distinguish it from that of most negative-strand RNA viruses. First, hantaviral genomic 5′ termini are processed to a monophosphate rather than retaining the 5′-triphosphate that constitutes the canonical RIG-I ligand, attenuating detection and delaying interferon induction [23]. Second, the cytoplasmic tail of Gn in pathogenic species inhibits RIG-I- and TBK1-directed interferon responses, an activity absent from nonpathogenic hantaviruses and therefore correlated with disease-causing potential [24]. It should be noted that the β3 integrins and protocadherin-1 discussed in Section 3 are entry receptors and not pattern recognition receptors; the two are occasionally conflated.

Following recognition, infected macrophages and dendritic cells secrete type I and type III interferons, IL-1β, TNF-α, IL-6, IL-15, and chemokines including CCL5 and CXCL10 [25]. Unlike many hemorrhagic fever viruses that impair dendritic cell maturation, hantavirus-infected dendritic cells mature normally and prime robust T cell responses, amplifying the adaptive immune response [26]. A sustained elevation in proinflammatory cytokines and plasminogen activation system components has been documented throughout the clinical course of SNV-associated HCPS [27].

CD8+ cytotoxic T lymphocytes reach peak expansion at the time of maximum clinical severity [26]. HLA typing studies support their pathogenic role: HLA-B35 is associated with increased disease severity and enhanced susceptibility to apoptosis, while HLA-B27 is associated with milder outcomes [28]. Virus-specific CD8+ T lymphocytes directed against SNV nucleocapsid protein epitopes have been isolated from acutely ill patients, producing IFN-γ and demonstrating cytolytic activity ex vivo [29]. The proposed mechanism of endothelial injury is cytotoxin and cytokine release in proximity to infected endothelial cells, impairing endothelial integrity through paracrine signaling.

A 2024 study demonstrated that activated inflammatory monocytes and macrophages are the immune subset most strongly correlated with disease severity in human HFRS and rodent models, driving a TNF-α-centered cytokine storm [30]. Plasminogen activator inhibitor-1 (PAI-1) rises 30- to 100-fold in plasma of terminal-stage SNV-infected patients, representing profound fibrinolysis inhibition and contributing to microvascular obstruction [31].

IFN-γ, the sole type II interferon, is distinct from the type I and type III interferons described above in its cellular source, its receptor (IFNGR1/IFNGR2), and its signaling output (JAK1 and JAK2 with STAT1 homodimers acting at GAS elements, rather than the ISGF3 complex). This distinction is temporally meaningful in HPS: the type I and III response is an early, cell-intrinsic antiviral program in infected endothelium and epithelium, whereas IFN-γ derives from NK cells, CD8+ cytotoxic T lymphocytes, and Th1 cells recruited later, and therefore coincides with the cardiopulmonary phase. TNF-α, IFN-γ, and nitric oxide released by these effectors increase capillary endothelial permeability and are proposed mediators of myocardial depression [26]. The interferon response in HPS is thus not a single entity but two temporally offset programs, the first largely protective and the second contributing to immunopathology.

Hantavirus-specific IgM antibodies are detectable at symptom onset in the majority of patients, reflecting an early plasmablast expansion [25,32]. IgG responses emerge concurrently and persist lifelong. Because seroconversion occurs during the long incubation period rather than after clinical deterioration, the appearance of specific antibody and the onset of the cardiopulmonary phase are temporally coupled; this underlies the utility of IgM serology as a diagnostic tool and is examined further in Section 6.2.

### 4.3. Hantaviral Myocarditis

Myocardial depression in HPS was originally attributed to cytokine-mediated contractile suppression. A histopathological study of 14 fatal HPS cases revised this interpretation: hantaviral antigen and viral particles were identified in cardiac endothelium and interstitial macrophages in all 14 cases, accompanied by discrete foci of myofiber necrosis, interstitial edema, and an inflammatory infiltrate of macrophages and memory T lymphocytes [33]. TNF-α expression was significantly greater in cardiac macrophages and cardiomyocytes in HPS patients than in controls dying of other causes with acute lung injury [33]. These findings establish hantavirus as a cause of myocarditis, though the relative contributions of direct viral injury and cytokine-mediated suppression to the clinical hemodynamic picture have not been quantified.

Transpulmonary thermodilution studies confirm reduced stroke volume index, reduced global ejection fraction, and reduced preload-related parameters alongside elevated EVLWI and PVPI [19]. Volume depletion from capillary leak reduces preload, and myocarditis with cytokine-mediated suppression reduces contractility, producing the mixed cardiogenic-distributive shock that defines advanced HPS. Lactic acidosis from the low cardiac output is among the strongest predictors of fatal outcome [34].

Thrombocytopenia occurs in 98% of patients, with platelet counts below 150 × 10^9^/L at presentation. Mechanisms include direct binding of pathogenic hantaviruses to platelet αIIbβ3 integrins, immune-mediated platelet destruction, and microvascular consumption [13,14]. Despite profound thrombocytopenia, fibrinogen levels are typically normal, distinguishing HPS coagulopathy from disseminated intravascular coagulation [17].

## 5. Clinical Presentation and Disease Phases

HPS advances through four phases: incubation, prodrome, cardiopulmonary, and diuretic-convalescent (Table 1). The incubation period averages 14 to 17 days, with a range of one to five weeks [35]. The prodromal phase lasts 3 to 10 days with fever, chills, myalgia, and gastrointestinal symptoms. Physical examination yields no specific findings, and the presentation is indistinguishable from nonspecific viral illness [34]. ANDV infection additionally produces facial flushing, fine petechiae, and conjunctival injection, features not typical of SNV disease [36].

The cardiopulmonary phase begins abruptly, typically within 12 to 48 h of prodromal onset. The interval from onset of dyspnea to need for mechanical ventilation is 1 to 6 h in some reported series [26]. Most patients develop hypotension within 24 h. Chest radiographs progress from peribronchial haze and Kerley B lines to diffuse bilateral alveolar infiltrates [19]. Fatal infections are marked by progressive myocardial depression evolving to sinus bradycardia, electromechanical dissociation, ventricular tachycardia, or fibrillation [34]. In survivors, the diuretic phase begins 2 to 4 days after peak illness, with polyuria and rapid resolution of pulmonary edema. Convalescence may take weeks to months.

## 6. Diagnosis

### 6.1. Clinical Recognition and Peripheral Blood Smear

Clinical recognition depends on epidemiological context (rural setting, rodent exposure, endemic region) combined with hematological findings [2]. A five-criterion peripheral blood smear triage protocol was established at the University of New Mexico Health Sciences Center: thrombocytopenia, hemoconcentration, granulocytic left shift, absence of toxic granulation, and immunoblasts exceeding 10% of lymphoid cells [37]. Retrospective validation over ten years (188 smear results compared with serology) confirmed that a four-of-five threshold yields sensitivity of 89% and specificity of 93% for serologically confirmed HCPS [38]. Meeting this threshold triggers preparation for critical care transfer and VA-ECMO. Because cytopenias may be absent during the prodrome, the complete blood count and smear should be repeated every 8 to 12 h in suspected cases.

### 6.2. Serological and Molecular Confirmation

ELISA for hantavirus-specific IgM is the preferred method for acute diagnosis. This may appear paradoxical in a disease in which death can follow within 24 to 48 h of the onset of the cardiopulmonary phase, and the reason it is not requires explanation. The incubation period of HPS is long, with a mean of 14 to 17 days and a range of one to five weeks [35], and seroconversion occurs during this interval rather than after clinical deterioration. Because the cardiopulmonary phase is substantially immune-mediated rather than a direct cytopathic consequence of viral replication, the appearance of specific antibody and the onset of clinical crisis are temporally coupled: hantavirus-specific IgM is detectable in the great majority of patients at the time of hospital presentation, with IgG frequently detectable concurrently [32,39]. Serological diagnosis therefore does not require knowledge of the time of exposure, since the operative reference point is symptom onset rather than infection. The clinically important exceptions are patients presenting very early in the prodrome and contacts of ANDV cases identified through person-to-person transmission, in whom viremia may precede detectable IgM; in these situations RT-PCR is the appropriate test, and we recommend that both be obtained concurrently (Section 9).

RT-PCR is accordingly indicated when patients present very early or when the diagnosis must be established before seroconversion. Suitable specimens include EDTA whole blood and nucleated blood cells or buffy coat, which generally yield higher sensitivity than serum or plasma because of cell-associated virus; fresh-frozen lung and other tissue at autopsy; and formalin-fixed tissue for immunohistochemistry where no antemortem specimen is available. Viral RNA is detectable in blood from the first 24 h of illness and for approximately ten days thereafter, and may be present before IgM and IgG appear [40]. Sensitivity declines through the cardiopulmonary phase as antibody titers rise [41], which is why RT-PCR functions as a complement to serology rather than a replacement for it. Most diagnostic assays target the S segment (N gene) for species-level identification; pan-hantavirus detection, including of novel or unexpected species, is generally performed with nested RT-PCR using degenerate primers directed at conserved RNA-dependent RNA polymerase motifs in the L segment [42]. No commercially manufactured, regulatory-cleared nucleic acid amplification test for hantavirus is currently available in the United States, and no single primer set is universally recommended. Confirmatory testing is performed at reference laboratories, in the United States by the Viral Special Pathogens Branch of the Centers for Disease Control and Prevention, using IgM and IgG μ-capture ELISA, strip immunoblot, RT-PCR, and immunohistochemistry [34]. Clinicians should contact their public health laboratory rather than assume local availability. Immunofluorescence and immunoblot assays are alternative confirmatory platforms [43].

### 6.3. Hemodynamic Thresholds

Plasma lactate above 4.0 mmol/L and cardiac index below 2.2 L/min/m^2^ at admission are independent predictors of fatal outcome [34]. Both parameters should be assessed at presentation and serially, as they define thresholds for VA-ECMO escalation. Hematocrit above 50% in men or 48% in women reflects severe intravascular depletion and is present in approximately 50% of cases [17]. Diagnostic and prognostic parameters are summarized in Table 2.

## 7. Management

### 7.1. Fluid and Hemodynamic Management

The central hemodynamic challenge in HPS is simultaneous intravascular volume depletion and permeability pulmonary edema. Fluid administration corrects hypovolemia but directly worsens pulmonary edema. Transpulmonary thermodilution data support conservative fluid strategies guided by EVLWI and PVPI [19]. No vasopressor or inotropic regimen has been validated in controlled trials of HPS, and cardiotonic agents are used to support depressed cardiac output [40].

High-volume hemofiltration (HVHF) has been reported in isolated cases as a strategy to remove accumulated extracellular fluid before refractory shock develops [44,45]. The evidence consists of case reports and small series; HVHF is a rational option in patients with progressive fluid overload who have not yet entered refractory cardiogenic shock but cannot be considered standard of care.

### 7.2. Mechanical Ventilation

Mechanical ventilation is required in most patients presenting in the cardiopulmonary phase. Lung-protective ventilation (tidal volume 6 mL/kg predicted body weight; plateau pressure ≤30 cmH_2_O) is applied by analogy with ARDS management, as no HPS-specific ventilatory trials exist [46]. Prone positioning has been used as bridge therapy to improve oxygenation during interhospital transfer to ECMO-capable centers [47].

### 7.3. Venoarterial ECMO

VA-ECMO supports both cardiac output and pulmonary gas exchange, bridging patients through the critical 24 to 72 h period of maximum capillary leak and myocardial depression. The CDC reports that VA-ECMO initiated at the earliest sign of cardiopulmonary decompensation is associated with an 80% survival rate [34]. An observational study of 47 HCPS patients in Chile (2015–2022) found overall cohort mortality of 33.6%, rising above 60% in patients meeting critical illness criteria who did not receive timely ECMO [48]. A 2024 review of Chilean HCPS experience reports that 25% of cases diagnosed in 2023 required VA-ECMO, with mortality of 43% to 76% in patients requiring mechanical ventilation and hemodynamic support before ECMO escalation [40].

Established criteria for VA-ECMO initiation are cardiac index below 2.2 L/min/m^2^, plasma lactate above 4.0 mmol/L, progressive lactic acidosis unresponsive to conventional support, or hemodynamically significant arrhythmia [34,40]. Interhospital transfer to an ECMO-capable center should be arranged before refractory shock develops.

### 7.4. Antiviral and Immunomodulatory Therapy

No antiviral agent has demonstrated efficacy in controlled trials for HPS. Intravenous ribavirin was evaluated in two clinical trials and showed no benefit in either; among fatal cases, most deaths occur within 24 to 48 h of the onset of the cardiopulmonary phase, which leaves a narrow window for any agent administered after hospital presentation [49,50].

Favipiravir inhibits SNV and ANDV in vitro at a 90% effective concentration at or below 5 μg/mL and significantly improves survival in the lethal ANDV Syrian hamster model when administered before viremia peaks [51]. Treatment initiated after viremia onset loses protective efficacy in this model. A 2021 in vitro study found comparable potency between ribavirin and favipiravir against Hantaan virus individually, with enhanced efficacy at reduced individual doses when combined [52].

High-dose intravenous methylprednisolone was evaluated in a double-blind, placebo-controlled randomized trial of 66 ANDV-associated HCPS patients in Chile. The composite primary endpoint was reached by 15 of 30 placebo-treated and 11 of 30 methylprednisolone-treated patients (*p* = 0.43), and no significant difference in mortality was observed between treatment groups (*p* = 0.41) [53]. The trial investigators concluded that although methylprednisolone appears to be safe, it did not provide significant clinical benefit and that their results do not support its use for HCPS [53]. Saúl et al. have noted that mortality was nonetheless lower in the treatment group than in the placebo group (27% vs. 40%), a difference that did not reach statistical significance and may reflect the small sample size [54]. Advantages of methylprednisolone include a short regimen (3 days) and the possibility of late administration (median 6 days). Glucocorticoids were used in 90.9% of 33 HCPS patients admitted to an ICU in Buenos Aires between 1999 and 2024, in whom overall mortality was 21.2% [55].

Recombinant human monoclonal antibodies targeting the ANDV Gn glycoprotein demonstrate potent neutralizing activity and protect against ANDV in the Syrian hamster model, providing a basis for future clinical evaluation [56].

### 7.5. Immune Plasma

Human immune plasma obtained through plasmapheresis of convalescent HCPS donors (≥6 months post-infection), with neutralizing antibody (NAb) titers quantified by focus-reduction neutralization test, was administered intravenously at a standardized dose of 5000 U/kg ANDV NAb to 29 confirmed HCPS cases [57]. This resulted in a 30-day case fatality rate of 14%, representing a reduction compared with untreated cases during the same 2008–2012 period (32%; *p* = 0.049, OR = 0.35, 95% CI 0.12–0.99), untreated cases at the same sites from 2005–2012 (27%; *p* = 0.15, OR = 0.43, 95% CI 0.14–1.34), and a concurrent methylprednisolone-treated cohort (33%; *p* = 0.052, OR = 0.32, 95% CI 0.10–1.00). No serious adverse events were associated with plasma infusion, and while post-transfusion NAb titers in recipients remained variable and viral load stable, these findings suggest that immune plasma is a safe and potentially efficacious therapeutic strategy for HCPS.

Despite these encouraging results, immune plasma has not entered routine use, for several reasons. The trial was open-label and non-randomized, and no randomized controlled trial has been conducted or is currently feasible given case numbers. Supply is the principal practical constraint: plasma must be obtained from convalescent donors with high neutralizing titers, a pool numbering in the tens of individuals per country, and requires plasmapheresis capacity in the rural and often remote regions where cases occur. Strain specificity compounds this, since neutralizing antibodies raised against ANDV and SNV do not cross-neutralize in vitro [58], so South American donor plasma is not expected to benefit North American patients, and no equivalent donor program exists in North America. The therapeutic window is narrow, as benefit is likely to depend on administration at or before the onset of the cardiopulmonary phase, whereas diagnosis is frequently made after decompensation. Finally, development has shifted toward defined products with more tractable supply characteristics: in addition to the recombinant monoclonal antibodies described in Section 7.4 [56], polyclonal human immunoglobulin produced in DNA-vaccinated transchromosomic bovines protects in lethal models of HPS [59]. None has yet been evaluated clinically in HPS.

### 7.6. Vaccine Development: Obstacles and Candidates

No licensed vaccine against New World hantaviruses exists, and the obstacles are as much structural as immunological. First, the epidemiology of HPS defeats conventional efficacy trials: cases are sporadic, geographically dispersed, and number fewer than 30 per year in the United States [5], so a placebo-controlled efficacy trial of feasible size cannot be adequately powered. Development therefore depends on immunobridging against a correlate of protection rather than on clinical endpoints. Second, the absence of a commercial market has left development largely dependent on biodefense funding. Third, animal models are limited: the Syrian hamster model of ANDV infection is essentially the only one that reproduces lethal HPS, and SNV causes no disease in hamsters, so SNV candidates cannot be tested against a disease endpoint at all. Fourth, antigenic diversity precludes a monovalent product, since neutralizing antibodies raised against SNV do not cross-neutralize ANDV or the reverse, so a pan-American vaccine must be multivalent [58]. Fifth, containment requirements restrict the number of laboratories able to work with these agents. Inactivated vaccines licensed regionally against hemorrhagic fever with renal syndrome, such as preparations used in the Republic of Korea and China, target Old World hantaviruses and confer no expected protection against HPS; their efficacy has in any case been questioned [60]. Candidates in development include M-segment DNA vaccines expressing Gn and Gc, which have undergone early-phase clinical evaluation [61], and newer nucleic-acid platforms employing prefusion-stabilized glycoprotein antigens [60].

## 8. Prognosis

Case fatality varies by virus, region, and access to critical care, and pooled estimates should be interpreted with that in mind. United States surveillance data spanning 1993 to 2023 give a case fatality rate of approximately 35% to 38% across 890 confirmed cases [1,5]. In Chile, where ANDV is the sole etiological agent, the historical rate is approximately 32% [57]. Across eight countries in the Americas during 2025, 229 cases and 59 deaths were recorded, a case fatality rate of 25.7% [6]. The apparent downward trend should be attributed cautiously; improved ascertainment of milder cases and expanded access to VA-ECMO both contribute, and neither represents a change in the intrinsic virulence of the agent.

Plasma lactate above 4.0 mmol/L and cardiac index below 2.2 L/min/m^2^ at presentation are the most reliable bedside predictors of death [34]. Multiorgan dysfunction is uncommon despite the severity of cardiopulmonary failure, which distinguishes HPS from other forms of viral septic shock. Survivors of the cardiopulmonary phase recover without permanent hepatic, renal, or neurological sequelae, though pulmonary function abnormalities have been documented in a minority [34].

## 9. Conclusions

HPS results from three converging mechanisms: increased microvascular permeability from αvβ3 integrin dysregulation and VEGF hypersensitivity; immunopathology from CD8+ T cells and macrophages releasing cytokines that amplify vascular leak, cause PAI-1-mediated coagulopathy, and suppress myocardial contractility; and direct hantaviral myocarditis causing structural cardiac injury. These processes generate a cardiogenic-distributive shock refractory to conventional resuscitation alone.

A practical management algorithm follows from the pathophysiology. When HPS is suspected on clinical and epidemiological grounds, a complete blood count with peripheral blood smear and hantavirus IgM serology with concurrent RT-PCR should be obtained immediately; because seroconversion precedes the cardiopulmonary phase, IgM will be positive in the great majority of patients at presentation, while RT-PCR covers those presenting during the prodrome. A four-of-five smear criterion score or a positive IgM result warrants ICU admission regardless of initial hemodynamic status. In the ICU, fluid resuscitation should be conservative, guided by EVLWI and PVPI on transpulmonary thermodilution, targeting correction of hypovolemia without worsening pulmonary edema. Serial plasma lactate and cardiac index should be measured at admission and every four to six hours thereafter. Contact with an ECMO-capable center should be initiated at the first appearance of any of the following: plasma lactate above 4.0 mmol/L, cardiac index below 2.2 L/min/m^2^, refractory hypotension, hemodynamically significant arrhythmia, or rapid oxygenation failure. Transfer should not await cardiac arrest; VA-ECMO initiated before circulatory collapse is associated with substantially better survival [34,48].

Clinical management rests on three pillars: early recognition during the prodromal phase using the peripheral blood smear triage protocol; hemodynamic monitoring by transpulmonary thermodilution to guide fluid balance; and early VA-ECMO at first hemodynamic decompensation. No antiviral has been proven effective in the cardiopulmonary phase, and methylprednisolone was shown to be ineffective in a randomized controlled trial. Research priorities include antiviral treatment during the prodromal phase before viremia peaks, VEGF-VEGFR2-targeted permeability interventions, definitive evaluation of neutralizing antibody products, and expansion of VA-ECMO infrastructure in endemic regions.

## Figures and Tables

**Table 1:** Clinical phases of hantavirus pulmonary syndrome.

Phase	Duration	Key Clinical Features	Key Laboratory Findings
**Incubation**	1–5 weeks (mean 14–17 days)	Asymptomatic. Seroconversion typically occurs during this phase.	None; hantavirus-specific IgM becomes detectable toward the end of the phase.
**Prodrome**	3–10 days	Fever, myalgia, headache, nausea, vomiting, diarrhea. ANDV: facial flushing, conjunctival injection, fine petechiae.	Early thrombocytopenia; circulating immunoblasts; mild transaminitis; normal or mildly elevated leukocyte count. Viremia detectable by RT-PCR.
**Cardiopulmonary**	Hours to 2–3 days; among fatal cases, most deaths occur within 24–48 h of the onset of this phase	Cough, dyspnea, hypoxia, bilateral alveolar infiltrates, pleural effusions, hypotension, tachycardia, cardiogenic shock, arrhythmia.	Thrombocytopenia (<150 × 10^9^/L in 98%); left-shift leukocytosis; hemoconcentration; immunoblasts >10%; elevated lactate; cardiac index <2.2 L/min/m^2^. IgM detectable in the great majority of patients.
**Diuretic/Recovery**	2–4 days diuresis; convalescence weeks to months	Polyuria, resolution of pulmonary edema, normalizing hemodynamics.	Normalizing platelet count, resolving hemoconcentration.

Abbreviations: ANDV, Andes virus.

**Table 2:** Diagnostic and prognostic parameters in hantavirus pulmonary syndrome.

Parameter	Threshold/Finding	Pathophysiological Basis	Clinical Significance
Platelet count	<150 × 10^9^/L (98% of cases); rapid progressive decline	Platelet αIIbβ3 integrin binding; immune-mediated destruction; microvascular consumption	Progressive thrombocytopenia marks transition to cardiopulmonary phase; serial monitoring required
Hematocrit	>50% (men); >48% (women); present in ~50% of cases	Massive capillary leak and intravascular fluid loss	Marker of severe capillary leak; correlates with disease severity
Plasma lactate	>4.0 mmol/L	Tissue hypoperfusion from low cardiac output	Independent predictor of fatal outcome; threshold for VA-ECMO consideration
Cardiac index	<2.2 L/min/m^2^	Cytokine-mediated myocardial depression and direct hantaviral myocarditis	Independent predictor of fatal outcome; threshold for VA-ECMO consideration
EVLWI and PVPI	Elevated; EVLWI inversely correlated with GEF (r = −0.36) and MAP (r = −0.27)	Permeability pulmonary edema from increased microvascular permeability	Guides fluid management and VA-ECMO timing; obtained by transpulmonary thermodilution
Peripheral smear immunoblasts	>10% of lymphoid series; sensitivity 89%, specificity 93% in validated series	CD8+ T cell and plasmablast expansion at peak immune activation	Part of validated five-criterion triage protocol; triggers VA-ECMO preparation
Hantavirus IgM	Detectable in the great majority of patients at hospital presentation	Seroconversion during the long incubation period precedes the immune-mediated cardiopulmonary phase	Preferred test for acute diagnosis; does not require knowledge of exposure timing
Hantavirus RNA by RT-PCR	Detectable from first 24 h to approximately day 10 of illness	Peak viremia during prodrome and early cardiopulmonary phase	Complements serology; test of choice in very early presentation and in traced ANDV contacts
PAI-1	30–100-fold elevation in terminal-stage patients	Inhibition of fibrinolysis; hemostatic imbalance	Elevated in severe HCPS; fibrinogen typically normal, distinguishing from DIC
sRAGE	Elevated in severe vs. mild HCPS	Type I alveolar epithelial cell injury	Emerging biomarker of alveolar epithelial injury; requires prospective validation

Abbreviations: ANDV, Andes virus; DIC, disseminated intravascular coagulation; EVLWI, extravascular lung water index; GEF, global ejection fraction; MAP, mean arterial pressure; PAI-1, plasminogen activator inhibitor-1; PVPI, pulmonary vascular permeability index; sRAGE, soluble receptor for advanced glycation end-products; VA-ECMO, venoarterial extracorporeal membrane oxygenation.

## Data Availability

No new data were created or analyzed in this study. Data sharing is not applicable to this article.

## Supplementary Materials

The following supporting information can be downloaded at https://www.mdpi.com/article/10.3390/v18080915/s1, Table S1: Basis for Reference Inclusion.
