# Supplementary material for: Cardiopulmonary Failure in Hantavirus Disease: Mechanisms, Recognition, and ECMO-Based Management
Source: Viruses. 2026 Aug 20;18(8):915. doi: 10.3390/v18080915 (PMC13517502; doi:10.3390/v18080915)
Supplement: Supplementary file 1 [file viruses-18-00915-s001.zip › viruses-4427364-supplementary.pdf]

Supplementary Table S1. Basis for Reference Inclusion.

Inclusion criteria as defined in Section 2 (Methods): (i) original human data on the pathophysiology, clinical course, diagnosis or treatment of HPS/HCPS; (ii) randomised or otherwise controlled trial; (iii) mechanistic experimental work bearing directly on microvascular permeability, immune response or myocardial injury; (iv) autopsy or histopathological series; (v) surveillance report, outbreak investigation or clinical guidance from a national or international public health authority; (vi) review cited only where it supplies a synthesis not available from primary sources.

Ref.	Source	Type of evidence	Basis for inclusion	Section(s) in which cited
1	MacNeil et al., Emerg Infect Dis 2011	National surveillance analysis	(v) Surveillance report from a national public health authority; source of US case counts and case fatality rate	1. Introduction; 8. Prognosis
2	Duchin et al., N Engl J Med 1994	Clinical case series (n=17)	(i) First systematic description of the clinical syndrome; primary source for presenting features	1. Introduction; 6.1. Clinical Recognition and Peripheral Blood Smear
3	Jonsson et al., Clin Microbiol Rev 2010	Review	(vi) Cited only for taxonomy and genome organisation, for which it is the standard synthesis and no single primary source is adequate	1. Introduction
4	Padula et al., Virology 1998	Outbreak investigation with molecular typing	(i, v) Primary molecular evidence for person-to-person ANDV transmission; no later source supersedes it	1. Introduction
5	CDC, Reported Cases of Hantavirus Disease	Surveillance database	(v) Official case counts used for US epidemiology and for the argument on trial feasibility in Section 7.6	1. Introduction; 7.6. Vaccine Development: Obstacles and Candidates; 8. Prognosis
6	PAHO, Epidemiological Alert, Dec 2025	Surveillance alert	(v) Most recent regional case and death counts for the Americas	1. Introduction; 8. Prognosis
7	Figueiredo et al., Virus Res 2014	Review	(vi) Cited for South American reservoir ecology, where it consolidates dispersed primary literature	1. Introduction
8	Goodfellow et al., PLoS Pathog 2025	Original field and laboratory study	(iii) Current primary evidence on reservoir host range and shedding	1. Introduction
9	Jääskeläinen et al., J Med Virol 2007	Mechanistic laboratory study	(iii) Primary demonstration that the NSs ORF product antagonises interferon-beta induction	1. Introduction
10	Andes Virus Outbreak Working Group, N Engl J Med 2026;394(24):2477-9	Outbreak report with clinical detail	(i, v) Primary clinical account of the MV Hondius cases, including presentation, differential diagnosis, and laboratory confirmation by pan-hantavirus RT-PCR and L-segment sequencing	1. Introduction
11	WHO, Disease Outbreak News (DON611), 2 July 2026	Closing outbreak report from an international authority	(v) Definitive final case and death counts, case fatality ratio, age distribution, transmission hypothesis, and contact-tracing figures for the 2026 outbreak	1. Introduction
12	ECDC, Threat Assessment Brief, 6 May 2026 (doi:10.2900/6502463)	Risk assessment from an international authority	(v) Independent assessment of transmission risk and of contact identification and management in the same outbreak	1. Introduction
13	Gavrilovskaya et al., PNAS 1998	Mechanistic laboratory study	(iii) Primary identification of beta-3 integrins as the entry receptor for pathogenic New World hantaviruses	3. Viral Entry and Cellular Tropism; 4.3. Hantaviral Myocarditis
14	Gavrilovskaya et al., J Virol 1999	Mechanistic laboratory study	(iii) Extends receptor usage to Old World hantaviruses; needed for the pathogenic/nonpathogenic contrast	3. Viral Entry and Cellular Tropism; 4.3. Hantaviral Myocarditis
15	Jangra et al., Nature 2018	Mechanistic laboratory study	(iii) Primary identification of protocadherin-1 as an entry factor explaining pulmonary tropism	3. Viral Entry and Cellular Tropism
16	Zaki et al., Am J Pathol 1995	Autopsy series	(iv) Primary evidence that endothelial replication occurs without cytopathic injury	3. Viral Entry and Cellular Tropism; 4.1. Increased Microvascular Permeability and Pulmonary Edema
17	Nolte et al., Hum Pathol 1995	Autopsy and pathological series	(iv) Primary source for haematological findings and the absence of endothelial necrosis	4.1. Increased Microvascular Permeability and Pulmonary Edema; 4.3. Hantaviral Myocarditis; 6.3. Hemodynamic Thresholds
18	Gavrilovskaya et al., J Virol 2008	Mechanistic laboratory study	(iii) Primary demonstration of VEGF hypersensitivity and its suppression by angiopoietin-1 and S1P	4.1. Increased Microvascular Permeability and Pulmonary Edema
19	López et al., Ann Intensive Care 2019	Prospective human haemodynamic study	(i) Only transpulmonary thermodilution dataset in HCPS; underpins the mixed-shock argument and fluid strategy	4.1. Increased Microvascular Permeability and Pulmonary Edema; 4.3. Hantaviral Myocarditis; 5. Clinical Presentation and Disease Phases; 7.1. Fluid and Hemodynamic Management
20	Meza-Fuentes et al., 2023	Cross-sectional human biomarker study	(i) Primary data on sRAGE as a marker of alveolar epithelial injury in HCPS	4.1. Increased Microvascular Permeability and Pulmonary Edema
21	Lee et al., 2011	Mechanistic laboratory study	(iii) Primary evidence for RIG-I as the principal sensor of hantaviral RNA	4.2. Innate Recognition and Immunopathogenesis
22	Handke et al., J Immunol 2009	Mechanistic laboratory study	(iii) Primary evidence for the TLR3 contribution to innate sensing	4.2. Innate Recognition and Immunopathogenesis

Ref.	Source	Type of evidence	Basis for inclusion	Section(s) in which cited
23	Habjan et al., PLoS One 2008	Mechanistic laboratory study	(iii) Primary evidence for 5'-monophosphate processing and attenuated RIG-I detection	4.2. Innate Recognition and Immunopathogenesis
24	Alff et al., J Virol 2006	Mechanistic laboratory study	(iii) Primary evidence that the Gn cytoplasmic tail of pathogenic species inhibits interferon induction	4.2. Innate Recognition and Immunopathogenesis
25	Saavedra et al., 2021	Review	(vi) Cited for the cytokine and chemokine profile, where it aggregates numerous small primary studies	4.2. Innate Recognition and Immunopathogenesis
26	Hallin et al., Crit Care Med 1996	Clinical case series	(i) Primary source for the tempo of cardiopulmonary deterioration and terminal rhythm disturbances	4.2. Innate Recognition and Immunopathogenesis; 5. Clinical Presentation and Disease Phases
27	Simons et al., 2021	Longitudinal human cohort	(i) Primary longitudinal cytokine and plasminogen-system data across the clinical course	4.2. Innate Recognition and Immunopathogenesis
28	Terajima et al., 2011	Review	(vi) Cited for the comparative immunopathogenesis of HPS and HFRS	4.2. Innate Recognition and Immunopathogenesis
29	Ennis et al., Virology 1997	Human immunological study	(i, iii) Primary isolation of virus-specific CD8+ T lymphocytes from acutely ill patients	4.2. Innate Recognition and Immunopathogenesis
30	Ma et al., 2024	Human and animal mechanistic study	(iii) Most recent primary evidence implicating inflammatory monocytes and macrophages in severity	4.2. Innate Recognition and Immunopathogenesis
31	Bondu et al., 2018	Human mechanistic study	(i, iii) Primary source for the magnitude of PAI-1 elevation in terminal disease	4.2. Innate Recognition and Immunopathogenesis
32	Bharadwaj et al., J Infect Dis 2000	Human serological study	(i) Primary data on IgM and IgG kinetics underpinning the diagnostic argument in Section 6.2	4.2. Innate Recognition and Immunopathogenesis; 6.2. Serological and Molecular Confirmation
33	Saggioro et al., Clin Infect Dis 2007	Autopsy and histopathological series (n=14)	(iv) The primary evidence establishing hantaviral myocarditis; central to Section 4.3	4.3. Hantaviral Myocarditis
34	CDC, Clinician Brief: HPS	Clinical guidance from a national authority	(v) Source of the ECMO survival figure and of the lactate and cardiac index thresholds	4.3. Hantaviral Myocarditis; 5. Clinical Presentation and Disease Phases; 6.2. Serological and Molecular Confirmation; 6.3. Hemodynamic Thresholds; 7.3. Venoarterial ECMO; 8. Prognosis; 9. Conclusions
35	Young et al., Am J Trop Med Hyg 2000	Epidemiological study	(i) Primary determination of the incubation period, on which the serology argument depends	5. Clinical Presentation and Disease Phases; 6.2. Serological and Molecular Confirmation
36	Toro et al., Emerg Infect Dis 1998	Outbreak investigation	(i, v) Primary description of ANDV-specific clinical features not typical of SNV disease	5. Clinical Presentation and Disease Phases
37	Koster et al., Am J Clin Pathol 2001	Diagnostic accuracy study	(i) Original derivation of the five-criterion peripheral blood smear protocol	6.1. Clinical Recognition and Peripheral Blood Smear
38	Dvorscak & Czuchlewski, Am J Clin Pathol 2014	Diagnostic validation study	(i) Ten-year validation giving the sensitivity and specificity quoted in Section 6.1	6.1. Clinical Recognition and Peripheral Blood Smear
39	Hjelle et al., J Clin Microbiol 1997	Diagnostic accuracy study	(i) Primary evidence on IgM detectability at presentation	6.2. Serological and Molecular Confirmation
40	Ulloa-Morrison et al., J Crit Care 2024	Narrative review	(vi) Cited for contemporary Chilean critical care practice and outcome figures not published elsewhere	6.2. Serological and Molecular Confirmation; 7.1. Fluid and Hemodynamic Management; 7.3. Venoarterial ECMO
41	Hjelle et al., J Virol 1994	Molecular virological study	(iii) Primary source on assay performance across the cardiopulmonary phase	6.2. Serological and Molecular Confirmation
42	Klempa et al., Emerg Infect Dis 2006	Methodological and virological study	(iii) Source of the degenerate nested L-segment RT-PCR approach for pan-hantavirus detection	6.2. Serological and Molecular Confirmation
43	Feldmann et al., Virus Res 1993	Laboratory study	(iii) Primary description of the nucleocapsid antigen used in confirmatory immunoassays	6.2. Serological and Molecular Confirmation
44	López et al., J Med Virol 2021	Small clinical series	(i) Best available evidence on high-volume haemofiltration; explicitly presented as non-definitive	7.1. Fluid and Hemodynamic Management
45	Bugedo et al., Rev Bras Ter Intensiva 2016	Case report	(i) Retained as the only other published account of haemofiltration in HCPS	7.1. Fluid and Hemodynamic Management
46	ARDS Network, N Engl J Med 2000	Randomised controlled trial	(ii) Cited as the basis for applying lung-protective ventilation by analogy, no HPS-specific trial existing	7.2. Mechanical Ventilation

Ref.	Source	Type of evidence	Basis for inclusion	Section(s) in which cited
47	Cornejo et al., Case Rep Crit Care 2013	Case report	(i) Retained for the specific management point of prone positioning during transfer to an ECMO centre	7.2. Mechanical Ventilation
48	Hernandez et al., Eur Respir J 2022	Observational cohort (n=47)	(i) Largest cohort addressing the timing of ECMO initiation; central to the review's main argument	7.3. Venoarterial ECMO; 9. Conclusions
49	Mertz et al., Clin Infect Dis 2004	Randomised placebo-controlled trial	(ii) Definitive negative trial of intravenous ribavirin in HPS	7.4. Antiviral and Immunomodulatory Therapy
50	Chapman et al., Antivir Ther 1999	Open-label trial	(ii) Complements ref. 49 on the safety and tolerance of ribavirin	7.4. Antiviral and Immunomodulatory Therapy
51	Safronetz et al., Antimicrob Agents Chemother 2013	Animal model study	(iii) Primary efficacy data for favipiravir and for the timing dependence of antiviral effect	7.4. Antiviral and Immunomodulatory Therapy
52	Mayor et al., Microorganisms 2021	In vitro study	(iii) Primary comparative potency data for ribavirin and favipiravir	7.4. Antiviral and Immunomodulatory Therapy
53	Vial et al., Clin Infect Dis 2013	Randomised double-blind controlled trial	(ii) The only randomised trial of corticosteroids in HCPS; primary source for the endpoint data quoted	7.4. Antiviral and Immunomodulatory Therapy
54	Saúl et al., Medicina (B Aires) 2021	Review and commentary	(vi) Cited to represent the published dissenting interpretation of ref. 53	7.4. Antiviral and Immunomodulatory Therapy
55	Cunto et al., Medicina (B Aires) 2025	Retrospective ICU cohort	(i) Contemporary real-world data on corticosteroid use and ICU mortality	7.4. Antiviral and Immunomodulatory Therapy
56	Garrido et al., Sci Transl Med 2018	Animal model study	(iii) Primary evidence for protective monoclonal antibodies against ANDV	7.4. Antiviral and Immunomodulatory Therapy; 7.5. Immune Plasma
57	Vial et al., Antivir Ther 2015	Non-randomised multicentre trial	(ii) The only prospective clinical evaluation of immune plasma; primary source for all figures in Section 7.5	7.5. Immune Plasma; 8. Prognosis
58	Valdivieso et al., Emerg Infect Dis 2006	Human immunological study	(iii) Primary evidence that SNV and ANDV antisera do not cross-neutralise, underpinning both the plasma-supply and vaccine-multivalency arguments	7.5. Immune Plasma; 7.6. Vaccine Development: Obstacles and Candidates
59	Hooper et al., Sci Transl Med 2014	Animal model study	(iii) Primary evidence for polyclonal transchromosomic bovine IgG as a defined alternative to donor plasma	7.5. Immune Plasma
60	Tscherne et al., npj Viruses 2026	Review	(vi) Cited for the current ANDV vaccine and therapeutic pipeline, which no primary source covers	7.6. Vaccine Development: Obstacles and Candidates
61	Hooper et al., Clin Microbiol Infect 2014	Phase 1 clinical trial	(ii) Primary evidence on early-phase clinical evaluation of M-segment DNA vaccines	7.6. Vaccine Development: Obstacles and Candidates
